# Alpha-1 antichymotrypsin is involved in astrocyte injury in concert with arginine-vasopressin during the development of acute hepatic encephalopathy

**DOI:** 10.1371/journal.pone.0189346

**Published:** 2017-12-07

**Authors:** Jonghyuk Park, Takahiro Masaki, Yoshihiro Mezaki, Hiroshi Yokoyama, Mariko Nakamura, Haruka Maehashi, Takahiko J. Fujimi, Sabine S. Gouraud, Keisuke Nagatsuma, Madoka Nakagomi, Naofumi Kimura, Tomokazu Matsuura

**Affiliations:** 1 Department of Laboratory Medicine, The Jikei University School of Medicine, Minato-ku, Tokyo, Japan; 2 Faculty of Health and Nutrition, Bunkyo University, Chigasaki, Kanagawa, Japan; 3 Department of Biology, Faculty of Science, Ochanomizu University, Bunkyo-ku, Tokyo, Japan; 4 Hatano Research Institute, Food and Drug Safety Center, Hadano, Kanagawa, Japan; 5 Department of Pharmacology, The Jikei University School of Medicine, Minato-ku, Tokyo, Japan; Nihon University School of Medicine, JAPAN

## Abstract

**Background and aims:**

We developed a bio-artificial liver (BAL) using a radial-flow bioreactor and rescued mini-pig models with lethal acute liver failure (ALF). The point of the rescue is the recovery from hepatic encephalopathy (HE). HE on ALF has sometimes resulted in brain death following brain edema with astrocyte swelling. Several factors, including ammonia and glutamine, have been reported to induce astrocyte swelling and injury. However, many clinicians believe that there are any other factors involved in the development of HE. Therefore, the aim of this study was to identify novel HE-inducible factors, particularly those inducing astrocyte dysfunction.

**Methods:**

Mini-pig plasma samples were collected at three time points: before the administration of toxins (α-amanitin and LPS), when HE occurred after the administration of toxins, and after treatment with extracorporeal circulation (EC) by the BAL. To identify the causative factors of HE, each plasma sample was subjected to a comparative proteome analysis with two-dimensional gel electrophoresis and mass spectrometry. To assess the direct effects of candidate factors on the astrocyte function and injury, *in vitro* experiments with human astrocytes were performed.

**Results:**

Using a proteome analysis, we identified alpha-1 antichymotrypsin (ACT), which was increased in plasma samples from mini-pigs with HE and decreased in those after treatment with EC by BAL. In *in vitro* experiments with human astrocytes, ACT showed growth-inhibitory and cytotoxic effects on astrocytes. In addition, the expression of water channel protein aquaporin-4, which is induced in injured astrocytes, was increased following ACT treatment. Interestingly, these effects of ACT were additively enhanced by adding arginine-vasopressin (AVP) and were canceled by adding an AVP receptor antagonist.

**Conclusions:**

These results suggest that ACT is involved in astrocyte injury and dysfunction in concert with AVP during the development of acute HE.

## Introduction

Hepatic encephalopathy (HE) is a major clinical complication in patients with severe liver disease and refers to the reversible neuropsychiatric disorder observed in acute liver failure (ALF) [1, 2]. Rapidly progressive HE in patients with ALF is a clinical syndrome associated with cerebral edema; it leads to cytotoxic brain edema, increased intracranial pressure, brain herniation, and ultimately to death. Cytotoxic brain edema, which principally occurs due to astrocyte swelling, is the major neuropathological finding in ALF [1, 3, 4].

We previously reported that a mini-pig model of ALF induced by α-amanitin, a mushroom-derived poison, and lipopolysaccharide (LPS) ultimately died from marked cerebral edema with increased S-100β protein in plasma, a marker of astrocytic damage, whereas mini-pigs with induced ALF that were treated with extracorporeal circulation (EC) using a bio-artificial liver (BAL) were able to recover from lethal HE. The survival of the mini-pigs with ALF treated with BAL therapy is thought to be due to the elimination of unknown HE-inducing factors from the blood using the EC system [5]. Although the molecular basis for the neurological disorder in ALF remains to be fully elucidated, elevated blood and brain ammonia levels have been strongly implicated in ALF [6]. Ammonia plays a major role in the development of astrocyte swelling/brain edema in ALF and it has also been shown to correlate with the degree of HE [7–9]. Moreover, the symptoms of hyperammonemia, a prerequisite for the development of cerebral edema in HE, become more pronounced in the presence of arginine-vasopressin (AVP, syn. antidiuretic hormone ADH). Additionally, the induction of AVP was shown to exacerbate the ammonia-induced increase in cerebral blood flow and to hasten the development of brain edema in a model of ALF [10, 11].

In the brain, aquaporin-4 (AQP4) is bidirectional transport channel for water, which is abundantly expressed in the astrocyte foot processes. In astrocytes, the expression of AQP4 is strongly associated with the development of brain edema/astrocyte swelling [12]. Altered AQP4 has been shown to contribute to cerebral edema in various neurological conditions, including not only HE associated with ALF [13] but also focal traumatic brain injury (TBI) [14], ischemic stroke [15], influenza-associated encephalopathy [16], and Alzheimer’s disease (AD) [17]. In addition, AQP4 is not restricted to brain edema [18], and various studies have demonstrated its important role in astrocytic oncosis and cell death [19]. For example, AQP4 deletion can reduce brain swelling and improve neurological outcomes in models of cytotoxic cerebral edema [13, 20]. However, the pathogenic factors responsible for astrocyte swelling in HE are not completely understood. Thus, identifying the molecular targets for the functional regulation of astrocytes and elucidating their therapeutic potential is expected to have important theoretical and practical significance.

In the present study, we mainly aimed to identify novel HE-inducible factors, particularly those inducing astrocyte dysfunction and expression of AQP4. Using a proteome analysis, we identified alpha-1 antichymotrypsin (ACT) in mini-pig plasma and found it was increased under conditions of severe liver failure and decreased after EC with the BAL. Therefore, ACT was chosen for further study. We investigated whether ACT alone or in combination with AVP affects astrocyte dysfunction and the AQP4 expression in human astrocytes. Our study suggests that ACT (with AVP) plays an important role in astrocyte injury and dysfunction during the development of acute HE.

## Materials and methods

### Acute liver failure model and plasma collection

The experimental treatment and mini-pig plasma sample collection was carried out as described in our previous published paper [5]. Male mini-pigs (CSK-MS) weighing 10–15 kg were generous gifted by Chugai Pharmaceutical (Tokyo, Japan). During the experiment, the mini-pigs were allowed access to feed and water ad libitum. The pigs were maintained in a temperature-controlled room and housed in individual cages. To minimize potential pain and distress of mini-pigs, the mini-pigs were anesthetized by inhalation of 3–4% isoflurane before the administration of hepatic toxins, and were euthanized under inhalation with an overdose of isoflurane. Acute liver injury in mini-pigs was induced with a combination of α-amanitin (Calbiochem, Darmstadt, Germany) at a dose of 0.05 mg/kg and lipopolysaccharide (LPS; Sigma, St. Louis, MO, USA) at 1 μg/kg, dissolved in 10 ml of saline, injected via the splenic vein. After the administration of the toxins, mini-pigs were monitored every one-hour. We also fixed electrocardiographic electrodes to the chest wall and girdled the animal with a belt incorporating a transducer to detect respiratory movement. Blood samples were collected from a catheter cannulated in a cervical artery at three times points: before the administration of hepatic toxins (mushroom-derived α-amanitin and LPS), when cerebral edema occurred following acute hepatic encephalopathy, and after treatment with EC by the BAL for 6 hours. Plasma was obtained from each blood sample by using a plasma separator tube.

The protocol of animal experiments was created based on the Guidelines for the Proper Conduct of Animal Experiments of the Science Council of Japan and approved by the Institutional Animal Care and Use Committee of the Jikei University.

### Preparation of plasma samples and two-dimensional gel electrophoresis

Samples (130 μg) were dissolved in rehydration buffer (7 mol/L urea, 4% CHAPS, 60 mmol/L dithiothreitol (DTT), 0.5% immobilized pH gradient (IPG) buffer), and were subsequently applied on an 11-cm IPG strip gel (pH 3–11) for isoelectric focusing (IEF) using the IPGphor system (Amersham Biosciences, Uppsala, Sweden). During the first-dimension electrophoresis, the IEF of proteins was performed in five steps (rehydration for 12 h, 200 V for 1 h, 500 V for 1 h, 1000 V for 1 h and 8000 V for 9 h). The strip gels were equilibrated for 30–40 min with a series of DTT, iodoacetamide and sodium-dodecyl sulphate polyacrylamide gel electrophoresis (SDS-PAGE) running buffer containing disequilibrium buffer. During the second dimension electrophoresis, the equilibrated strip gels were run on a 12.5% polyacrylamide SDS gel using a PROTEAN II electrophoresis kit (Bio-Rad, Hercules, CA, USA).

### LC-MS/MS mass spectrometry

Liquid chromatography-tandem mass spectrometry (LC-MS/MS) was performed for protein identification. The separation column (0.2 x 50 mm, C18 AQ particles, 3 μ, 200 Å) was from Michrom BioResources. Peptides were separated in acetonitrile gradient (buffer A- 2% acetonitrile and 0.1% formic acid; buffer; B- 90% acetonitrile and 0.1% formic acid) with a flow rate of 1 μl/min using a Paradigm MS-4 HPLC system (Michrom BioResources, Inc., USA) and applied on-line to a LCQ (Thermo Finnigan, USA) ion-trap mass spectrometer. Peak lists were generated and calibrated using the Mascot software program (Matrix Science), and the MS/MS-based peptide was validated. The Mascot search parameters were as follows: two missed cleavages were permitted in trypsin digestion; the variable modifications included oxidation of methionine, propionamidation of cysteine, and phosphorylation of serine, threonine, and tyrosine; the peptide mass tolerance was ± 2 Da; the fragment mass tolerance was ± 0.8 Da.

### Cell line and culture conditions

A human astroglial cell line was purchased from the American Type Culture Collection (CRL-8621^TM^, SVG p12; ATCC, Manassas, VA, USA) and maintained in ASF104N medium (Ajinomoto, Tokyo, Japan) supplemented with 10% fetal bovine serum (FBS) (Atlas Biologicals, USA) or with 10% dialyzed FBS with an undetectable level of AVP (Biological industries, USA). Cells were incubated at 37°C in a humidified atmosphere 5% CO_2_ in air.

### DNA florescence intensity analyses using Hoechst 33342

To investigate whether or not ACT affects cell viability, astrocyte cells were moved into black 96-well culture plates (NUNC Optical bottom plate, Thermo Fisher Scientific, USA) at a density of about 1 × 10^4^ cells per well. ACT was added to the astrocytes at a concentration of 0.01–0.5 mg/ml for 24 h. Cells were counter-stained with Hoechst to assess the nuclear morphology. The Hoechst staining was performed by incubating the 96-well plates for 30 min at 37°C with 10 μg/ml Hoechst no. 33342 (Sigma) diluted in minimum essential medium (MEM; Gibco, USA) without glutamine or phenol red. The DNA fluorescence intensity measured by Skanlt RE Varioskan Flash 2.4 (Thermo Scientific, MA, USA) at 350 nm excitation and 460 nm emission.

### Treatment (cell stimulation)

The human astroglial cell line was cultured in 24-well plates and exposed to NH_4_Cl (5 mM), ACT (0.5 mg/ml), AVP (100 nM), and combination ACT and AVP for 6, 12, 24, and 48 h, respectively. The AQP4 protein expression in astrocytes was then analyzed by Western blotting, and the AQP4 expression was analyzed by quantitative reverse transcription polymerase chain reaction (qRT-PCR).

### Hoechst 33342/propidium iodide staining

The apoptotic morphology of human astrocytes was compared using the Hoechst 33342/propidium iodide dual staining method. In brief, the cells were cultured on poly-L-lysine-coated glass-bottom dishes (Matsunami Glass, Osaka, Japan) for an additional two to three days. The cells were collected and incubated with the fluorescent DNA-binding dyes Hoechst 33342 (10 μg/ml) and propidium iodide (PI) (10 μg/ml) for 20 min at 37°C. Nuclei were visualized using a BZ-8100 fluorescence microscope (Keyence, Osaka, Japan). Cell death percentages were calculated by determining the ratio of PI-stained dead cells to Hoechst 33342-stained cells (Hoechst 33342 stains the nuclei of all cells, dead or alive).

### Isolation of total RNA and real-time quantitative RT-PCR

The cells were collected, and the total RNA was extracted using Trizol reagent (Ambion) according to the manufacturer’s instruction. The amount of total RNA was measured using a Nano-Drop ND-1000 spectrophotometer. cDNA was generated from total RNA by reverse transcription (RT) using oligo (dT)12-18 primers (Invitrogen) and superscript RT (Invitrogen) in an Applied Biosystems 2720 Thermal Cycler (Applied Biosystems). The RT steps consisted of incubation at 65°C for 5 min followed by incubation at 50°C for 60 min and 75°C for 15 min. The RT products were used as templates for PCR amplification of 20-μL reactions. Real-time PCR was performed using the following: 10 μL of SYBR Green Mix, 0.4 μL of each primer, 2 μL of cDNA template, and 7.2 μL of dH_2_O. The mixture was incubated at 95°C for 10 s and 60°C for 1 min for 40 cycles, terminating at 95°C for 15 s, 60°C for 1 min, and 95°C for 15 s. Changes in the AQP4 expression were examined using a StepOnePlus real-time PCR system (Applied Biosystems).

### Western blot analyses

The human astrocytes cultured in 24-well pates were lysed in RIPA lysis buffer (Santa Cruz Biotechnology, Santa Cruz, CA, USA). After lysis for 10 min on ice, the cells were centrifuged at 12,000 rpm for 10 min, and supernatant was transferred to a new tube. Protein quantification was performed using a DC protein assay kit (Bio-Rad, Hercules, CA, USA). Samples for the analysis of AQP4 were heated to 95°C for 10 min. Cell lysates (10 μg) were separated by sodium dodecyl sulfate-polyacrylamide gel electrophoresis (SDS-PAGE) and transferred to a polyvinylidene difuoride membrane (Atto, Tokyo, Japan). Nonspecific binding was blocked by 5% skim milk phosphate-buffered saline (PBS) supplemented with 0.1% Tween 20 (Merck, Darmstadt, Germany) (PBST) for 1 h at room temperature. Membranes were incubated with a primary antibody against AQP4 (1:500, mouse monoclonal; Sigma) in PBST overnight at 4°C, washed with PBST, and incubated at room temperature for 30 min with a horseradish peroxidase-conjugated goat anti-mouse IgG secondary antibody (1:10,000; GE Healthcare Little Chalfont, Buckinghamshire, UK). After washing, the membrane was visualized using an enhanced chemiluminescence method (ECL; Amersham Biosciences, GE Healthcare Little Chalfont, Buckinghamshire, UK). The optical densities of the bands were measured with the ChemiDoc^TM^ Touch Imaging System (Bio-Rad). An internal control was used to ensure equal protein amounts in the sample (β-actin, 1:1,000, Sigma-Aldrich). These experiments were repeated at least three times, and representative data were used.

### Statistical analyses

Experimental data are expressed as the mean ± standard error of the mean. The statistical significance of the differences was assessed by Student’s *t*-test. A value of P < 0.05 was considered to be significant.

## Results

### Identification of the novel hepatic encephalopathy-inducible factors particularly inducing astrocyte dysfunction

**Fig 1:**
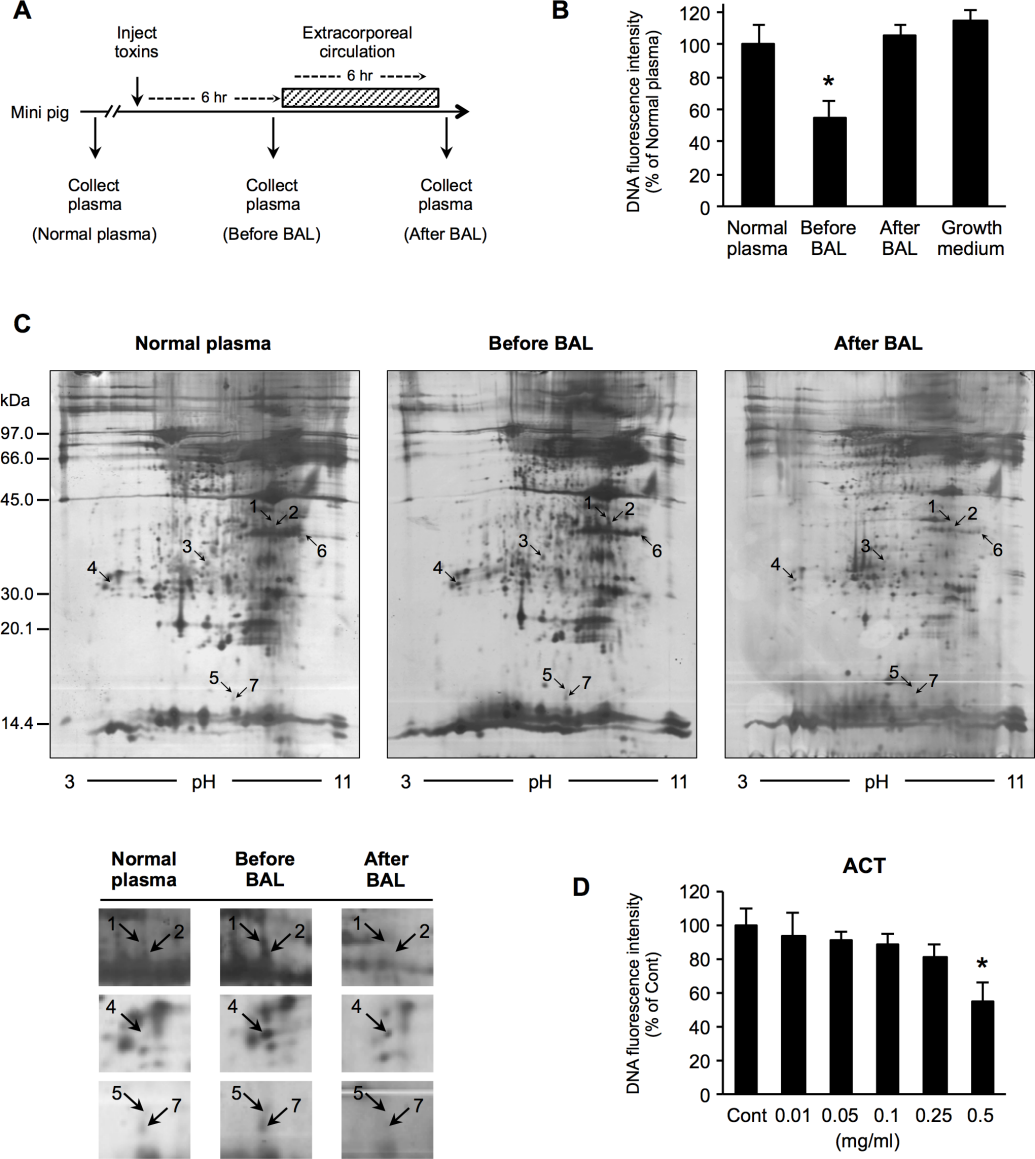
**The growth and viability of human astrocytes cultured with plasma from mini-pigs with HE and the identification of candidate causative factors for HE by a 2-DE-based proteomic analysis** (A) Experimental design: Mini-pig plasma was collected before the administration of α-amanitin and LPS, when encephalopathy occurred after the addition of toxins, and after treatment with extracorporeal circulation. (B) Astrocytes were cultured in medium with the collected plasma for 24 h. (C) Representative 2-DE gel images of silver-stained plasma proteins. These were based on LC-MS/MS data obtained from the NCBInr database using the MASCOT searching program. The labels 1 to 7 indicate the seven proteins that were upregulated before BAL treatment in comparison to normal plasma and which were downregulated after BAL treatment in comparison to before BAL treatment. (D) Astrocytes were incubated with various concentrations of ACT (0.01–0.5 mg/ml) for 24 h. Cell nuclei were counterstained with Hoechst 33342 with wavelengths of 350 nm excitation and 460 nm emission. Bars indicate the mean ± standard error of the mean (*n* = 5). *p < 0.05, in comparison to control (normal plasma).

As shown in Fig 1A, we collected plasma sample from a mini-pig at three times points: before the administration of the hepatic toxins, when cerebral edema following acute hepatic encephalopathy with ALF occurred, and after the treatment with EC by BAL. In the present study, we initially investigated whether or not the collected plasma had a cytotoxic effect on a human astrocyte cell line *in vitro*. Astrocytes were incubated in medium supplemented with mini-pig plasma for 24 h, and Hoechst 33342 was used to measure the DNA fluorescence intensity. In the plasma from the HE, the DNA fluorescence intensity of astrocytes was markedly decreased compared to normal plasma. However, after the BAL therapy, the DNA fluorescence intensity was similar to that in normal plasma (Fig 1B). The ammonia concentration of each plasma sample was 38 μg/dl in normal plasma, 114 μg/dl in plasma obtained before BAL, and 378 μg/ml in plasma obtained after BAL. Despite recovery from encephalopathy after EC through BAL therapy, the concentration of ammonia did not decrease. These results suggested that there was no association between recovery from acute HE and the blood ammonia concentration. We therefore attempted to identify the causative factors of HE using pig plasma samples. Each plasma sample was subjected to a comparative proteome analysis with two-dimensional gel electrophoresis and mass spectrometry, and peptide fragments derived from several proteins were found to be more abundant in plasma obtained after the development of HE than in those obtained after EC by BAL (Fig 1C). The proteome analysis identified several peptides of the same protein ACT as novel HE-inducing candidate factors upregulated before BAL treatment in comparison to normal plasma and downregulated after BAL treatment in comparison to before BAL treatment. Detailed information on these peptides is shown in Table 1. Among the proteins identified, we focused our subsequent analyses on ACT.

**Table 1:** Identification of candidate causative factors of HE with a 2-DE-based proteomic analysis.

Spot no.	Protein name	Accession no.	Mass	Score	Peptide matched	Sequence coverage (%)
1	α2-macroglobulin	gi|194307849	169371	460	20	8
	α1-antichymotrypsin	gi|194038347	46782	327	7	19
2	α2-macroglobulin	gi|194307849	169371	715	20	11
	α1-antichymotrypsin	gi|194038347	46782	255	9	16
3	α2-macroglobulin	gi|194307849	169371	151	4	3
	Unnamed protein product	gi|194384240	59042	115	7	6
4	rCG50690	gi|149031970	21249	136	6	16
	Hemoglobin subunit alpha	gi|122465	15087	83	3	17
5	α1-antichymotrypsin	gi|194038347	46782	132	5	7
6	α1-antichymotrypsin	gi|194038347	46782	378	14	17
	Similar to SERPINA3-6	gi|194038349	38357	317	13	14
7	α1-antichymotrypsin	gi|194038347	46782	199	4	7
	rCG50690	gi|149031970	21249	147	4	10

2-DE, Two-dimensional gel electrophoresis; Mass, molecular weight of a peptide. Spot number corresponds to the labels in Fig 1C. Protein names and accession numbers are listed according to the NCBInr database. The MASCOT probability-based MOWSE (molecular weight search) score was calculated for PMF. The score is -10*Log (*P*), where *P* is the probability that the observed match is a random event; a score of >49 was considered significant (*p*<0.05).

We then tested whether or not ACT treatment of astrocytes reduced the DNA fluorescence intensity. Astrocytes were treated with various concentrations of ACT (0.01 to 0.5 mg/ml) for 24 h. ACT treatment significantly reduced the DNA fluorescence intensity of astrocytes at 0.5 mg/ml, whereas low and middle concentrations did not (Fig 1D). These data suggested that increased ACT may be involved in the development of astrocyte disorder following HE.

### Cytotoxic effects in astrocytes treated with NH_4_Cl and ACT

**Fig 2:**
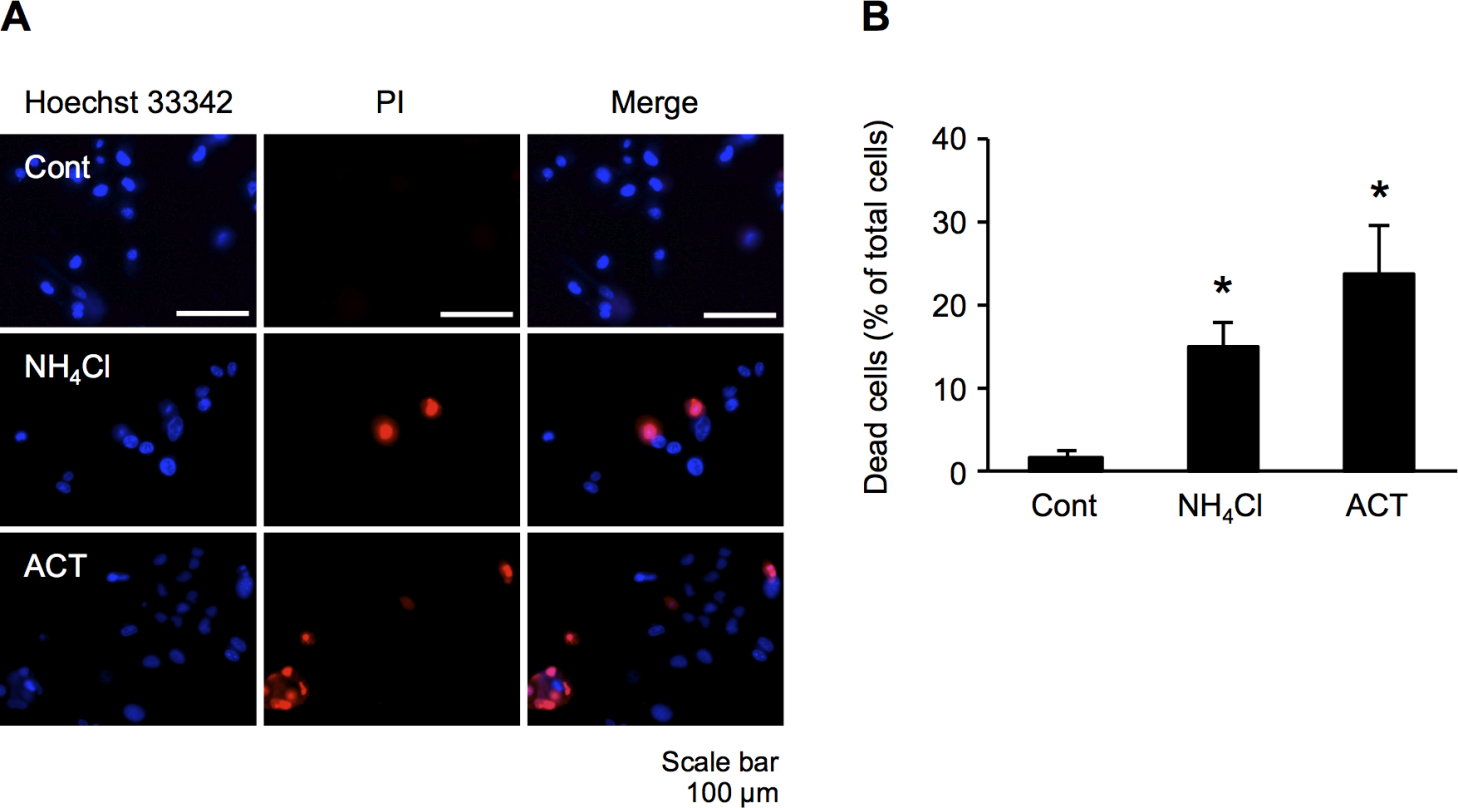
ACT induces astrocyte damage. (A) Astrocyte cells were co-stained with Hoechst 33342 and propidium iodide (PI). Cells were treated with NH_4_Cl (5 mM) and ACT (0.5 mg/ml) for 48 h. Final observation was conducted by fluorescence microscopy. (B) Percentages of cell death were calculated by determining the ratio of PI-stained cells to Hoechst-stained cells. Cells stained by PI represent dead cells, whereas Hoechst 33342 staining reveals all nuclei. Bars indicate the mean ± standard error of the mean (*n* = 3). *p < 0.05, in comparison to control.

To determine whether or not astrocytes treated with NH_4_Cl and ACT are prone to astrocyte damage, we assessed the nuclear morphological changes by Hoechst 33342/propidium iodide (PI) double staining. As shown in Fig 2A, the control astrocytes were stained blue (Hoechst 33342), and dead cells stained by PI were not observed. In contrast, astrocytes treated with NH_4_Cl and ACT for 48 h showed damage. PI staining showed that the number of dead cells was significantly increased in comparison to control after treatment with both ACT and NH_4_Cl (Fig 2B). However, the effects of treatment with ACT were not significantly different from the effects of treatment with NH_4_Cl. These results show that ACT and NH_4_Cl both have a cytotoxic effect on human astrocytes.

### Time course of AQP4, mRNA and protein expression in astrocytes after NH_4_Cl, ACT treatment

**Fig 3:**
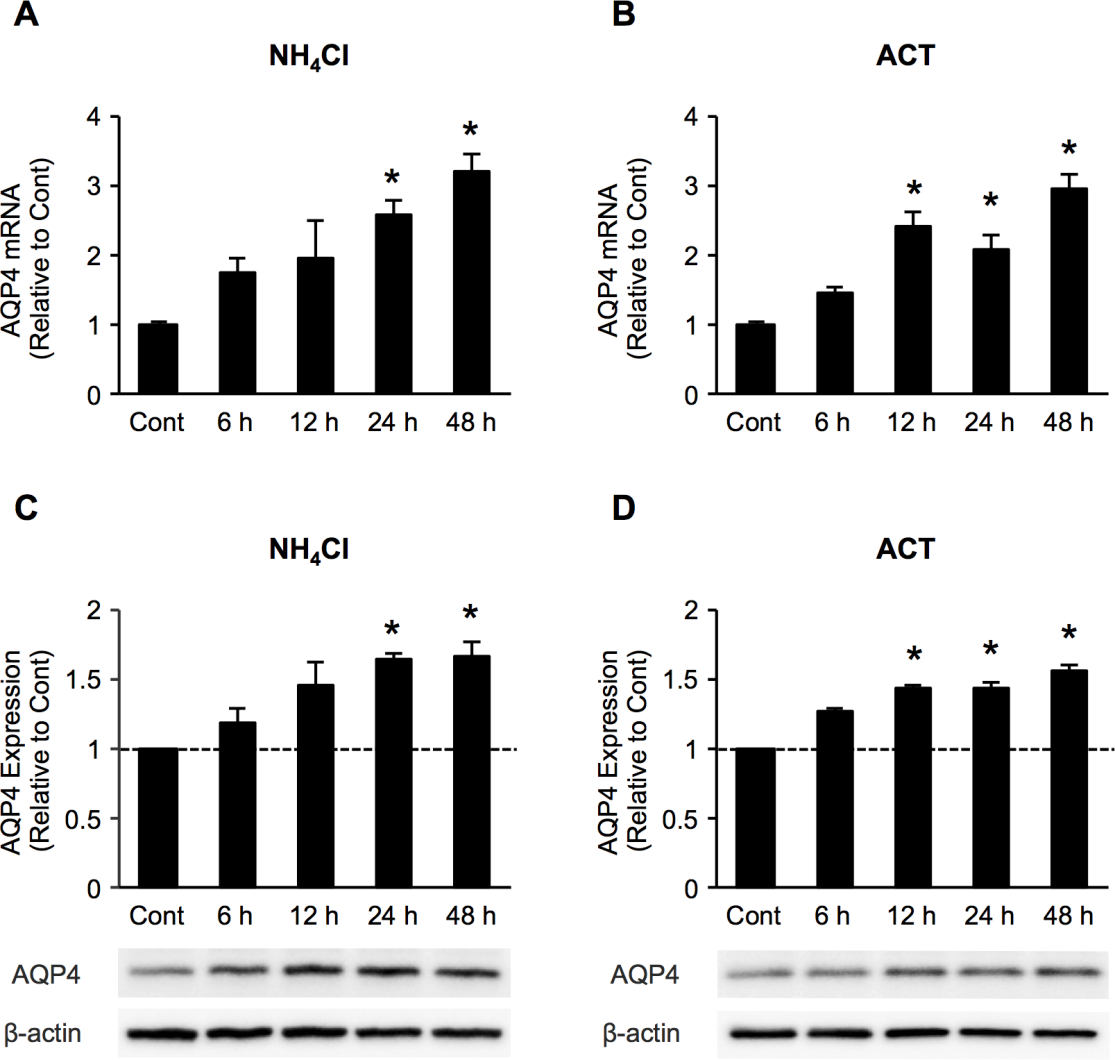
ACT increases AQP4 expression in astrocytes. Time course of the expression of AQP4 mRNA and protein in astrocytes at 6, 12, 24, and 48 h after treatment with NH_4_Cl and ACT, respectively. (A, B) The expression of AQP4 mRNA was analyzed by quantitative real-time RT-PCR and compared to the respective untreated controls. The expression, standardized by the level of GAPDH, is shown as folds of expression. (C, D) The AQP4 protein level was analyzed by Western blotting with an AQP4-specific monoclonal antibody. Band densities were analyzed using the Image Lab 5.1 software program (Bio-Rad) and are expressed as a percentage of the untreated control. Bars indicate the mean ± standard error of the mean (*n* = 3). *p < 0.05, in comparison to control.

Previous studies have reported a time-dependent increase in the AQP4 expression in cultured astrocytes exposed to a pathophysiological concentration of 5 mM ammonia (NH_4_Cl), which is correlated with the development of astrocyte swelling, leading to brain edema [21]. We therefore investigated the time-course change in the AQP4 mRNA expression in astrocytes after NH_4_Cl and ACT treatment by qRT-PCR. The mRNA expression of AQP4 was significantly increased after NH_4_Cl treatment at 24 and 48 h (Fig 3A). These results are consistent with those of previous reports [21, 22]. In order to determine the effects of ACT on AQP4 expression, astrocytes were treated with 0.5 mg/ml ACT for various periods of time. The AQP4 mRNA level was significantly increased at 12 h to 48 h after ACT treatment (Fig 3B). Consistent with the mRNA expression of AQP4, Western blotting showed that the AQP4 protein level was significantly increased in astrocytes following treatment with NH_4_Cl and ACT compared to controls (Fig 3C and 3D). These finding suggested that ACT might be involved in the upregulation of AQP4 expression in astrocytes.

### Effects of AVP and combination of ACT and AVP on astrocyte damage

**Fig 4:**
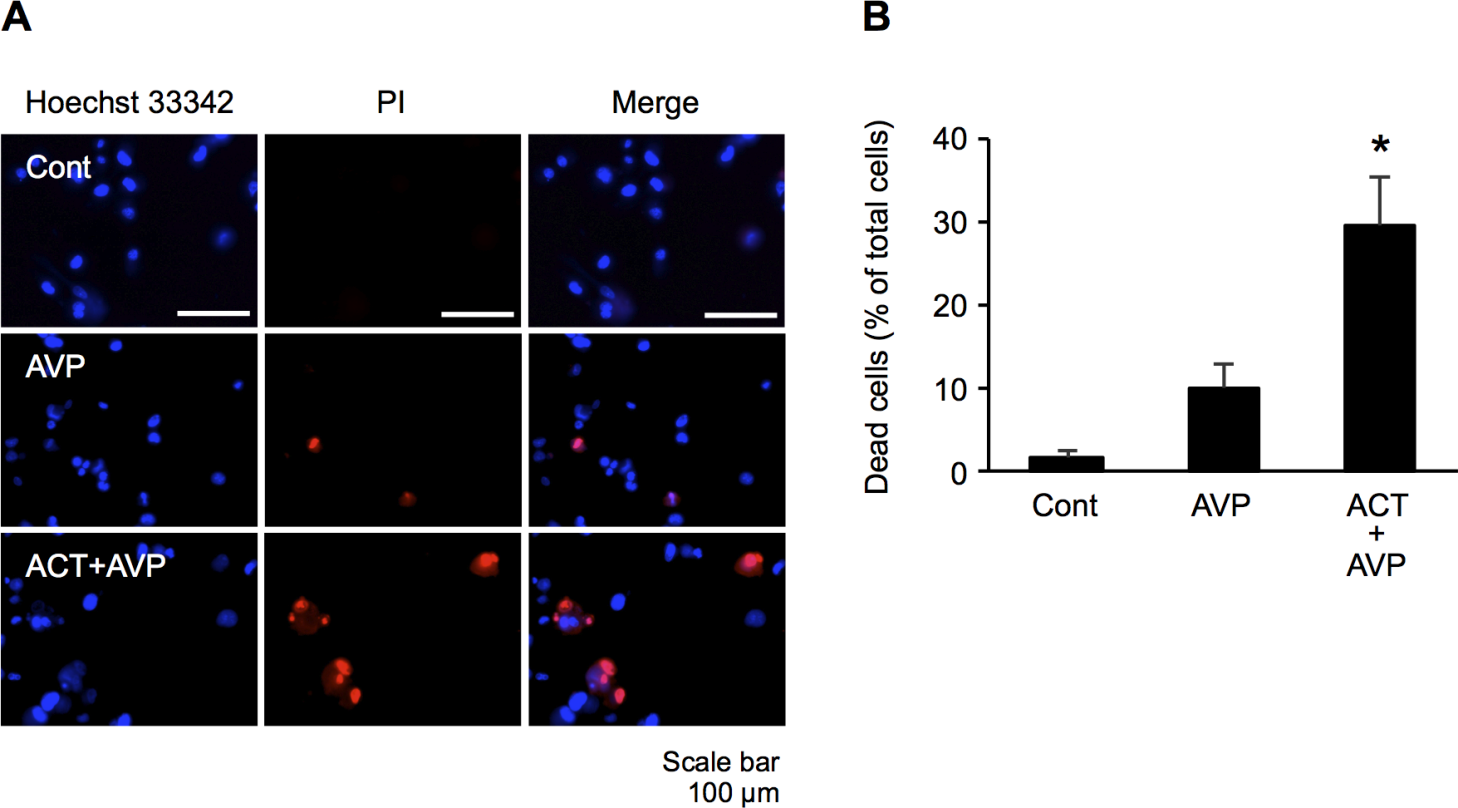
Effects of ACT and AVP on astrocyte damage. (A) Astrocyte cells were co-stained with Hoechst 33342 and PI. Cells were treated with AVP (100 nM) and combination with ACT (0.5 mg/ml) and AVP for 48 h. (B) Percentages of cell death were calculated by determining the ratio of PI-stained cells to Hoechst stained cells. Bars indicate the mean ± standard error of the mean (*n* = 3). *p < 0.05, in comparison to control.

During fulminant ALF, hyperammonemia leads further increases the level of AVP [11]. AVP has been suggested to play an important role in several brain pathologies that are associated with the formation of brain disorder and edema, including focal cerebral ischemia, intracerebral hemorrhage, and TBI. [23]. To investigate the effects of AVP on astrocyte damage, we tested whether or not AVP alone and in combination with ACT treatment induced astrocyte damage. PI staining showed significantly greater astrocyte damage following treatment with both ACT and AVP than with AVP alone, with no significant differences in damage between control cells and those treated with AVP alone, suggesting that ACT may act synergistically with AVP to induce astrocyte damage (Fig 4A and 4B).

### Expression of AQP4 in astrocytes treated with AVP alone and in combination with ACT

**Fig 5:**
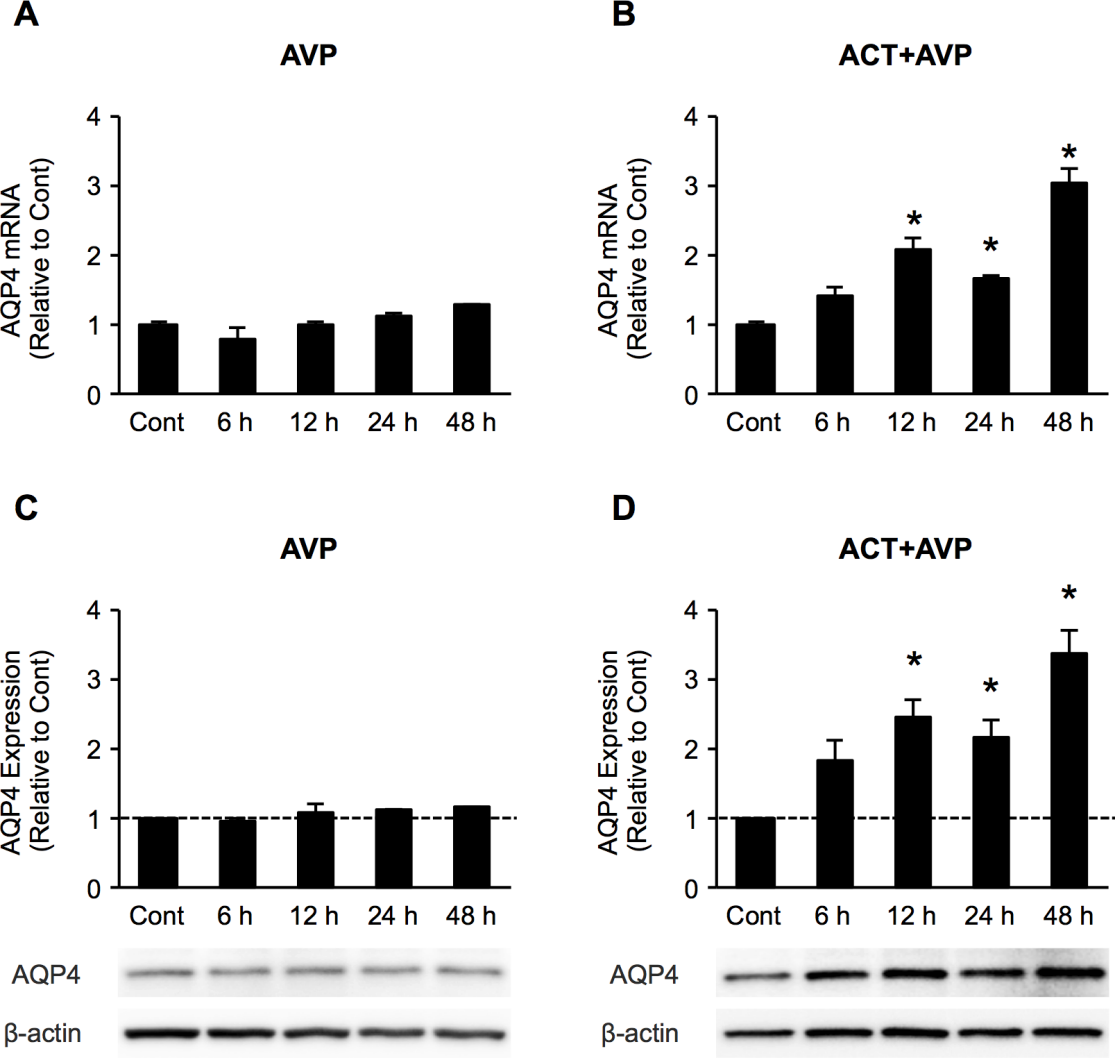
Effects of ACT and AVP on AQP4 expression in astrocytes. Time course of the expression of AQP4 mRNA and protein in astrocytes at 6, 12, 24, and 48 h after treatment with AVP and combination of ACT and AVP. (A, B) The expression of AQP4 mRNA was analyzed by quantitative real-time RT-PCR and compared to the respective untreated controls. The expression, standardized by the level of GAPDH, is shown as folds of expression. (C, D) The AQP4 protein level was analyzed by Western blotting. Bars indicate the mean ± standard error of the mean (*n* = 3). *p < 0.05, in comparison to control.

While AVP regulates AQP2 expression in the kidney through vasopressin V2 receptors, recent evidence has shown that brain AQP4 is modulated by AVP as well [24]. We investigated the effect of AVP on the induction of AQP4 expression when used in combination with ACT treatment. A time-course analysis of the AQP4 mRNA expression revealed that astrocytes treated with both ACT and AVP had significantly higher AQP4 levels than controls (Fig 5B), although there were no significant differences in the expression between control cells and those treated with AVP alone (Fig 5A). Western blotting showed that the AQP4 protein levels at 48 h were approximately 3.2-fold greater in astrocytes following treatment with ACT and AVP than in controls (Fig 5D), while treatment with AVP alone had no effect on AQP4 protein expression (Fig 5C). AVP therefore seems to accelerate the ACT-induced AQP4 protein expression in astrocytes.

### Effect of AVP1a receptor inhibitor on astrocyte damage and cellular AQP4 expression

**Fig 6:**
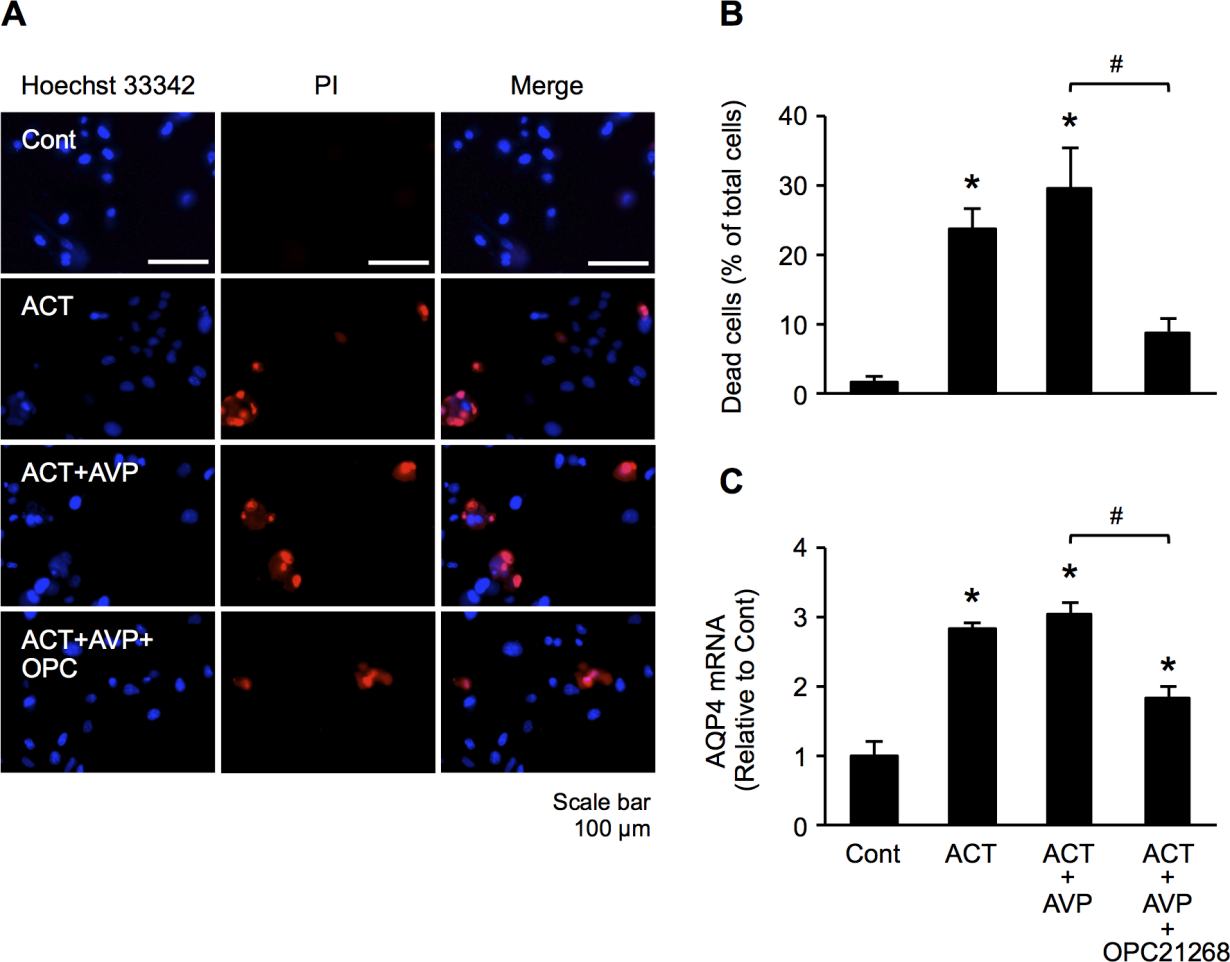
V1aR inhibitor reduces astrocyte damage and cellular AQP4 expression. (A) Hoechst 33342 and PI double-staining in astrocytes. Cells were treated with ACT, combination of ACT and AVP, and combination of ACT and AVP with OPC-21268 for 48 h. (B) Percentages of cell death were calculated by determining the ratio of PI-stained cells to Hoechst stained cells. (C) The expression of AQP4 mRNA at 48 h after ACT, combination of ACT and AVP, and combination of ACT and AVP with OPC-21268 were analyzed by quantitative real-time RT-PCR. Bars indicate the mean ± standard error of the mean (*n* = 3). *p < 0.05, in comparison to control, # *p* < 0.05.

**Fig 7:**
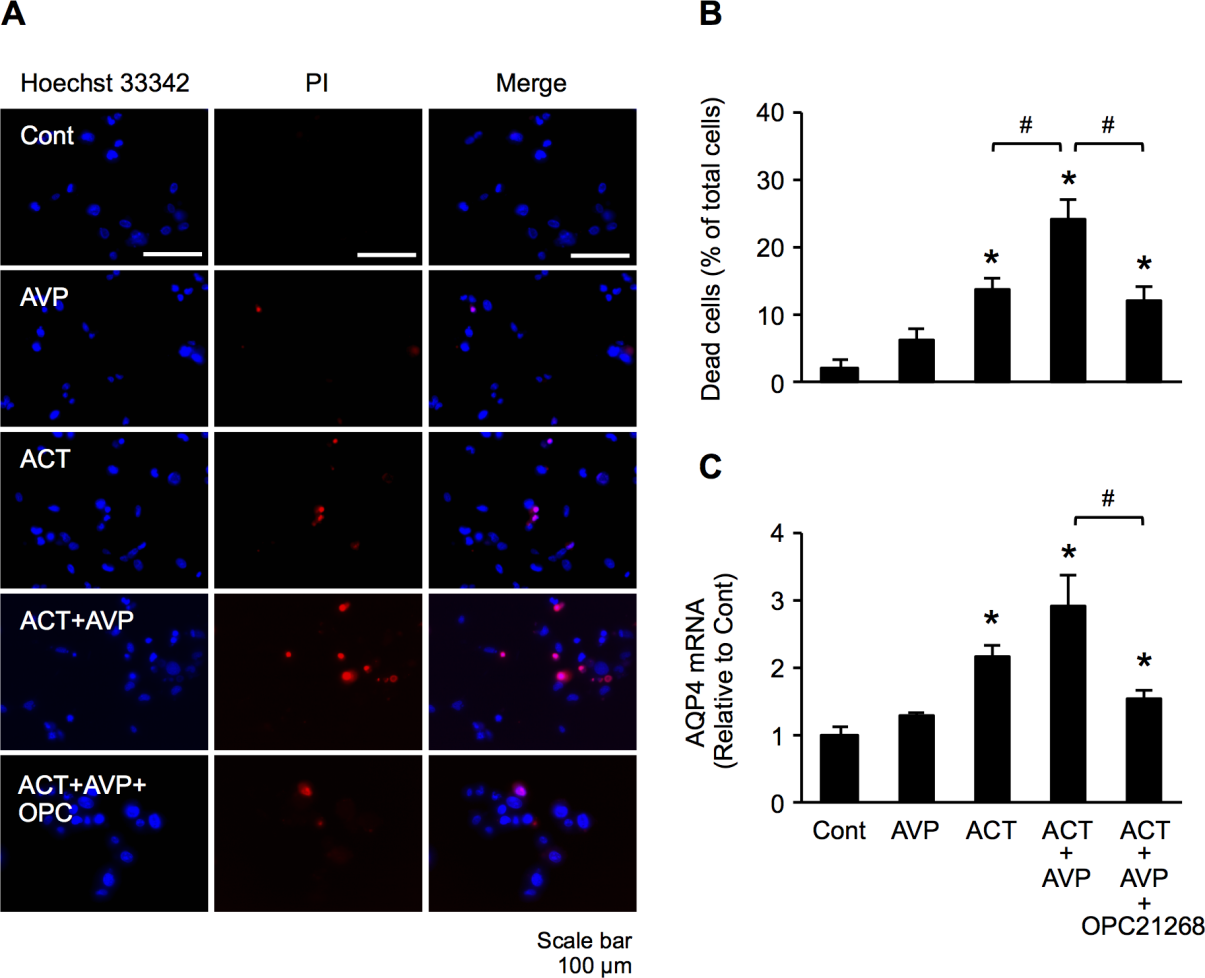
ACT and AVP additively induced astrocyte injury. (A) Hoechst 33342 and PI double-staining of astrocytes. Cells were cultured for 48h in AVP-free medium supplemented with AVP, ACT, a combination of ACT and AVP, or a combination of ACT and AVP with OPC-21268. (B) The percentages of dead cells were calculated by determining the ratio of PI-stained cells to Hoechst-stained cells. (C) The expression of AQP4 mRNA in cells cultured under the same conditions as (A) was analyzed by a quantitative real-time RT-PCR. The bars indicate the mean ± standard error of the mean (*n* = 3). *p < 0.05, in comparison to control, # *p* < 0.05.

AVP1a receptors (V1aR) are known to also be expressed within the brain, where they are suspected to play an important role in water homeostasis and in the evolution of brain edema [24]. There is growing evidence that brain V1aR modulates the expression and function of AQP4 in astrocytes [14]. We examined the effect of a V1aR inhibitor (OPC-21268) on both astrocyte damage and AQP4 expression. This V1aR inhibitor, at least in part, suppressed astrocyte damage induced by the treatment of ACT and AVP (Fig 6A and 6B). Similarly, ACT and AVP combination-induced AQP4 mRNA levels were also suppressed significantly compared to values in cells without V1aR inhibitor treatment (Fig 6C). These results suggest that the increased astrocyte damage and AQP4 expression observed after treatment with ACT and AVP may be, at least in part, mediated by V1aR in astrocytes. However, the effects of treatment with ACT alone or ACT plus AVP, with regard to astrocyte damage and the expression of AQP4 mRNA, did not differ to a statistically significant extent. Thus, we hypothesized that these results might have been due to the presence of a high concentration of endogenous AVP derived from the FBS in the culture medium. We therefore measured the concentration of AVP in culture medium supplemented with FBS (which we normally use). The concentration of AVP was 4.1 pg/ml. To make the effects of AVP clearer, we repeated the same experiments using cell culture medium supplemented with dialyzed FBS with an undetectable concentration of AVP. PI staining showed that the damage in cells treated with a combination of ACT and AVP was significantly greater than that in the control cells (Fig 7A and 7B). Astrocyte damage was induced by the addition of ACT alone, and AVP further enhanced the cytotoxic effect of ACT on astrocytes. However, OPC-21268 significantly inhibited this effect (Fig 7B), suggesting that AVP may be required for ACT to induce astrocyte injury. Although the AQP4 mRNA level was also significantly increased after treatment with the combination of ACT and AVP and by treatment with ACT alone, the mRNA expression of AQP4 did not differ to a statistically significant extent between the two treatment conditions. However, OPC-21268 significantly inhibited this effect (Fig 7C), suggesting that ACT may induce astrocyte injury in concert with AVP.

## Discussion

The pathophysiology of brain edema is complex and the critical factors that are responsible for acute HE after ALF remain controversial. [25] The main purpose of the present study was to identify novel HE-inducible factors, particularly those inducing astrocyte dysfunction and the expression of AQP4. The identification of biomarkers with proteomic technology has become an important tool in biomedical research [26]. A proteomic analysis remains one of the most common approaches for assessing severe hepatic diseases, such as mining cancer [27], hepatitis B [28] and C virus biomarkers, and hepatic cirrhosis [29]. However, proteins that are specifically regulated during acute HE have not been identified as diagnostic biomarkers of acute cerebral edema followed acute hepatic failure. We therefore used proteomic techniques to compare plasma proteomes between mini-pigs with HE in ALF and mini-pigs rescued by BAL therapy. A mass spectrometry analysis resulted in the identification of various proteins. Among them, ACT was identified in five spots as a candidate causative factor of HE in plasma collected from large animals. Moreover, α2-macroglobulin (α2M) was identified with ACT at several locations. One possible explanation for its involvement is because LPS induces the release of proinflammatory cytokines [30], as it has been reported that the *ACT* gene as well as α2M is heavily modulated by the presence of proinflammatory cytokines, including interleukin-1 (IL-1), IL-6, tumor necrosis factor-α (TNF-α) [31, 32].

ACT (referred to as SERPINA-3) is a serine protease inhibitor that is also known an acute phase protein that is chiefly synthesized in the liver and other tissues, including the lungs and brain. In the brain, it is mainly produced by activated astrocytes found near brain beta-amyloid (Aβ) deposits [33]. ACT is also associated with several fundamental biological processes, such as inflammation, blood coagulation, and apoptosis [34]. Most research into the role of ACT in disease thus far has focused on Alzheimer’s disease (AD) pathology and AD-associated neuropathological changes [35, 36]. For example, ACT protein in plasma and cerebrospinal fluid can induce astrocyte activation and enhance the ability of Aβ to activate astrocytes, inducing an array of potentially neurotoxic responses. A previous study revealed that the exposure of cultured mouse neurons to ACT (0.5 mg/ml) for 24 h resulted in an increased number of TUNEL-positive cells, indicating that these cells were undergoing apoptosis [34]. Astrocytes communicate with neurons directly and are usually more resilient than neurons after impairment; however, severe injury will cause astrocyte dysfunction, leading to increased neuronal death [19]. Although it is currently unknown whether or not ACT that is synthesized in liver is able to cross the blood-brain barrier (BBB), a previous study demonstrated a strong correlation between the CSF and plasma concentrations of ACT. This suggests that plasma ACT crosses the BBB and that ACT in CSF originates mainly from the blood [37]. We examined whether or not ACT might also affect the morphological changes in astrocytes. We used Hoechst 33342/PI staining to monitor the injury of astrocytes after treatment with ACT. We found that ACT treatment decreased cell proliferation and increased cell death in astrocytes. This result indicates that an increased ACT level might induce cytotoxic effects on cultured astrocytes.

AQP4 is widely accepted to play a key role in the cerebral edema process during the acute stage of HE [3, 38]. Furthermore, increased AQP4 is associated with not only astrocyte swelling, but also BBB damage, and cell death [20]. Current evidence indicates that brain AQP4 is involved in various astrocytic functions related to neurological diseases, including brain fluid and ion homeostasis [39]. In addition, the induction of AQP4 expression and edema formation has been well studied in animals with influenza-associated encephalopathy induced by proinflammatory cytokines, including IL-1, IL-6 and TNF-α in cultured astrocytes [16]. In the present study, we investigated whether or not ACT directly influences the expression of AQP4 in astrocytes. We found that ACT increases the expression of AQP4 mRNA and protein in a time-dependent manner, suggesting that ACT also plays an important role in the induction of AQP4 expression in astrocytes.

However, the symptoms of hyperammonemia, a prerequisite for the development of brain edema in fulminant ALF, become more pronounced in the presence of AVP. Furthermore, a few studies have shown that AVP and V1aR play crucial roles in the regulation of brain water and ion homeostasis, probably by modulating aquaporin-mediated flux through the astrocyte plasma membrane [14, 23, 24]. In the present study, however, the expression of AQP4 mRNA and protein in astrocytes was not affected by AVP alone. Interestingly, the combination of ACT and AVP markedly increased the AQP4 expression in a time-dependent manner compared to control cells and those treated with AVP alone. Western blotting showed that the AQP4 protein levels at 48 h were approximately 3.2-fold greater in astrocytes treated with both ACT and AVP than in those treated with ACT alone. A previous study demonstrated that AVP infusion alone, while causing a modest increase in the cerebral blood flow, does not lead to brain swelling; brain edema only occurs on exposure to the combination of AVP and ammonia. These findings suggest that AVP may potentiate an ammonia-induced increase in brain water and intracranial pressure in ALF model and hasten the development of brain edema [10, 11].

Furthermore, OPC-21268, a non-peptide V1aR antagonist, reduced the brain tissue levels of water and electrolytes following cold lesion brain injury [14, 40, 41]. The interaction between V1aR inhibition and AQP4 associated with astrocyte injury has been studied in models of ischemia stroke [15], intracerebral hemorrhaging [42], and TBI [14]. In a model of TBI, a selective V1aR antagonist prevented brain edema, and reduced astrocyte swelling and the expression of AQP4 [14]. The results indicate that V1aR inhibition reduces the AQP4 expression and water flux in the brain. In the present study, astrocyte injury and the AQP4 mRNA expression were markedly enhanced following treatment with ACT alone or the combination of ACT and AVP. However, under these two conditions, the astrocyte damage and the mRNA expression of AQP4 did not differ to a statistically significant extent (Fig 6). This suggests that the endogenous AVP in FBS reduced the impact of AVP supplementation. In order to make the effects of AVP clearer, astrocytes were cultured in medium supplemented with AVP-free FBS. The effect of ACT was additively enhanced by the addition of AVP and was canceled by adding a V1a receptor antagonist, suggesting that ACT may act synergistically with AVP to induce astrocyte injury.

In conclusion, we identified ACT as a candidate causative factor of cerebral edema following acute HE and showed that ACT exerted growth-inhibitory and cytotoxic effects on astrocytes. In addition, the expression of AQP4 was found to be increased following ACT treatment. To our knowledge, no studies have demonstrated any relationship between ACT and the expression of AQP4 associated with HE. These effects of ACT were potentiated by AVP and canceled by adding an AVP receptor antagonist, suggesting that ACT might be a therapeutic target and a potent plasma biomarker for acute hepatic encephalopathy. However, whether or not similar effect will be induced in *in vivo* experiments is unclear at present. Further studies will contribute to a better understanding of the role of ACT in astrocyte injury during the development of lethal hepatic encephalopathy on ALF.
